# Diphtheria serology in adults in Central Java and East Java, Indonesia: the importance of continuous diphtheria vaccination

**DOI:** 10.4314/ahs.v21i3.23

**Published:** 2021-09

**Authors:** Sunarno, Febriyani Asri, Subangkit, Mursinah, Herna, Susanti Nike, Sariadji Kambang, Widoretno, Febriyana Dwi, Febrianti Tati, Saraswati Ratih Dian, Puspandari Nelly

**Affiliations:** Centre for Research and Development of Biomedical and Basic Health Technology, National Institute of Health Research and Development, Ministry of Health, Jakarta, Indonesia

**Keywords:** Adult, diphtheria, Indonesia, serology

## Abstract

**Background:**

Vaccination increase immunity against diphtheria, yet will decrease by aging. Therefore, boosters are needed to be done regularly.

**Objectives:**

This study aims to determine the immunity to diphtheria for the population of 16 years old and above.

**Methods:**

The sample of study were 295 collected blood serums by Riskesdas project in 2013, the criteria was above 15 years of age and originating from the Provinces of Central Java or East Java inclusively. Immunity assessment was based on antibody titer (IgG) against diphtheria using Vero Cell cytotoxicity test. Statistical analysis was performed using the X2 test.

**Results:**

The full protective IgG titer (>0.1 IU/ml) at the age of 16–20 years included 75% sample with a geometric mean titer (GMT) of 0.19 IU/ml. Yet, at the age of 21–60 years and > 60 years, full protective IgG titers only cover 45.5% and 33.3% sample with GMT respectively 0.06 IU / ml. Statistical analysis showed the relationship between age and immune status with p-value 0.003. Otherwise, no relationship between the status of immunity with sex and residency with p-values of 0.16 and 0.43.

**Conclusions:**

The immune status against diphtheria at the age of above 15 years decreases with aging.

## Introduction

By 2020, the whole world is facing a major health problem, the pandemic Covid 19. However, other health problems must not be ignored, including diphtheria. Diphtheria is one of deadly disease that can be prevented by vaccination. The global case fatality rate (CFR) is around 10%, and keep increasing in severe cases^1^,^2^. Since diphtheria vaccination is widespread, the incidence of disease has decreased dramatically. However, several sporadic and outbreak events continue to occur in various countries. Indonesia, in this case, is the country with the 2^nd^ or 3^rd^ of the most diphtheria cases in the world recently. WHO data shows the number of diphtheria cases in Indonesia over the past 5 years, those were 495 cases (2019), 1026 cases (2018), 954 cases (2017), 342 cases (2016), and 529 (2015). Case fatality rate (CFR) of diphtheria in Indonesia about 5% of the total cases^3^–^6^.

Vaccination policies for diphtheria may vary between countries. According to Ministry of Health, the policy for diphtheria vaccination in Indonesia requires 7 times at the age of 2 months – 3 months – 4 months – 18 months followed by at the 1st grade of elementary school, and then 2nd and 5th grade. The vaccination are mostly carried out with combined vaccines. There were pertussis, tetanus, haemophilus influenza b and hepatitis B (DPT-HB-Hib/pentavalent) vaccines. Repetition (booster) is done with DPT-HB-Hib vaccine (age 18 months), DT (grade 1 primary school) and Td (grade 2 and 5 elementary school)^7^–^9^.

Based on annual report, coverage of DTP3 in Indonesia is quite high, it is 82% in average since 2011 to 2015^7^. In 2016, the coverage is increase significantly (93.3%) but it tend to decrease in 2017 and 2018 (88.3% and 85.49%).^3^,^5^,^6^ Even though, the National Basic Health Surveillance (Riskesdas) data, with different approach have shown that DTP3 coverage in Indonesia was lower than the annual report data^10^. The high coverage of immunization will enforce on decreasing number of the circulation of disease agents in the environment indirectly. It is well known that a decrease in the circulation of diphtheria-causing bacteria can reduce the potential for natural maintenance of immunity against diphtheria obtained from the environment. Immunity to diphtheria will decrease with increasing age. It could trigger diphtheria outbreaks if it is not anticipated, as happened in Russia and surrounding areas in the 1990s^7^,^11^. Therefore, most European countries have adopted the policy for providing immunization against diphtheria every 10 years to maintain immunity to remain high and protective. The policy against diphtheria has also been recommended in the United States and other countries^12^,^13^. However, until now the policy has not been taken by the Indonesian government, so it is necessary to assess the immune status of diphtheria in the adult population.

Immunity against diphtheria can be assessed from a person's antibody titer (IgG). IgG titers <0.01 IU/ml are considered non-protective or seronegative. IgG titers 0.01 - 0.09 IU/ml are considered partial protective. Whereas IgG titer >0.1 IU/ml is considered as full protective^14^. Examination of antibody titer (IgG) against diphtheria can be done with several methods, such as in vivo with experimental animals (gold standard), Vero Cell cytotoxicity (alternative gold standard), and ELISA^15^. Indonesia, the National Institute of Health Research and Development, Ministry of Health, has conducted the a nationwide survey to assess the immunity of the population against diphtheria in Riskesdas project in 2007 and 2013, but is still limited to under 15 years of age. This study aims to obtain a figure of immunity against diphtheria at the age of above 15 years, then it will be compared to immunity at the age of <15 years based on the results of previous studies.

## Method

### Sample

The samples of the study were 295 stored blood serums collected in Riskesdas project 2013^16^. The samples were selected based on the criteria of people with over 15 years of age and originated from the Provinces of Central Java or East Java inclusively. Those samples were selected because this study aims to evaluate the level of immunity in adults. The samples were based on residency, the respondents were Central Java and East Java residents. East Java and Central Java are neighboring regions on the Java Island, but they both disclosed a contrast number of reported cases. There were no complete data about the history of diphtheria vaccine administration, history of suffering or contacting with diphtheria cases because the study usd archive sampel with limited data.

### Vero cell cytotoxicity test (Neutralization testing)

Measurement of IgG titer against diphtheria was carried out by the neutralization method as an alternative gold standard testing the level of immunity against diphtheria. Vero cell neutralization is a method for determining the functionality of antibodies against diphtheria. The procedure followed the steps as per the WHO guidelines^15^. The working arrangement consisted of: preparation of extraction buffer for vero cell inspection; Complete Medium preparation for Vero cells; Culture and Preparation of Vero cells; Determine the dose of diphtheria toxin; MTT Assay / Formazan Extraction; Minimum Cytopathic Dose Diphtheria Toxin Calculation; Titration of immune sera (Vero cell toxin neutralization test); and Calculation of relative antibody titers. The minimum essential medium (MEM) was prepared by adding bovine serum (final concentration of 5–10%), L-glutamine (2 mM), D-glucose (0.1%), HEPES (0.015 M), penicillin (100 U/ml) and streptomycin (100 µg/ml). Vero cells were prepared to be nearly 4 × 105 cells/ml concentration in complete medium. Diphtheria toxin (Sigma) used a dose of 4x minimum cytopathic dose (MCD) which was determined by titration.

For the examination of toxin neutralization, serial two-fold dilutions (column 1–11) test samples (A-G) and as a control, anti-diphtheria toxin (H) were performed along complete medium in 96-well tissue culture microplate. As an internal control, anti-diphtheria toxin (Biofarma) standards were titrated on each plate. Control cells were placed in column 12 (12A-12D), while toxins controls were placed in column 12 (12E-12H). After diphtheria toxin (4 × MCD) had been added to all wells (except for control cells), the mixtures were incubated at room temperature for 1 hour. Vero cells had been dropped into the plate, before they were tightly sealed and incubated at 37ºC in 5% CO_2_ incubator for 6 days. After 6 days, the MTT assay were used to determine end points. The plates were visually observed for discoloration and absorbance with OD 550–570 nm on the microplate reader. The presence of dark blue color indicating viable cells, because mitochondrial dehydrogenase in viable cells was capable to reduce MTT into a colored formazan product. Whereas, light blue color indicating partial toxicity and the absence of color change indicating complete toxicity and dead cell.

End-points were recorded as scores based on dilution of the test sample and control anti-diphtheria toxin in the last well of the viable cells (OD value> 50% control OD). The first end-point well of sample was written with a score of 1, and so multiplied by 2 until the endpoints well at well 11 are recorded with a score of 1024. End-point scores were converted into a relative titer by comparing the end point control of the anti-diphtheria toxin on each plate. The end-point titer in the sample test were then calculated by multiplying the sample endpoint score with control, the antidiphtheria toxin endpoint. Those test were invalid and had to be repeated if there was no toxicity in (“toxin control”) and / or no growth in “control cell” and / or no end-point for control, the anti-diphtheria toxin.

### Statistical Analysis

The samples were divided into 3 groups, based on age. They were 16–20 years old (young adults), 21–60 years old (adults), and above 60 years old (elderly). Samples were also analyzed based on gender and sample origin. There were physiological differences and activities between men and women which could affect the immune response and the level of exposure to disease-causing agents in the environment. Antibody titers (IgG) are divided into 3 groups, based on seronegative or non-protective level (<0.01 IU/ml), partial protective (0.01 - 0.09 IU/ml), and full protective (> 0.1 IU/ml) as a marger of full protective (0.1 - 0.9 IU/ml) and long term protective (> 1 IU/ml) (14). Geometric mean titers (GMT) were calculated by Microsoft Excel 2010. The statistical significance of the differences was analyzed by X2 with a p-value <0.05 was considered significant.

## Results

### Samples and antibody titer

**Figure 1:**
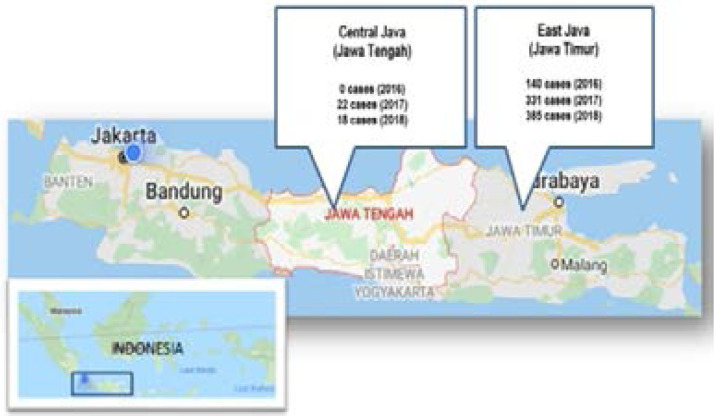
Map of Central Java n East Java along with the number of reported diphtheria cases (3,5,6). (photos were modified from the google map screenshot).

The provinces (respondent's residency) selected for sampling of this study were based on the closeness of the region, yet the significant difference in the reported number of diphtheria cases can be seen in Figure 1. Figure 1 shows that geographically the two provinces are neighbors and there are no strict boundaries, except the governance. However, the number of diphtheria cases is significantly different between those two. In this study, it could be seen whether the differences in immune status against diphtheria were associated or not between Central Java and East Java. Samples that fit the criteria are drawn proportionally by province, not by district.

Characteristics of the sample based on age, sex, and sample origin can be seen in Table 1. Table 1 also shows the proportion of the sample based on immune status as assessed by antibody titer (IgG) against diphtheria, which is not protective (< 0.01 IU/ml), partial protective (0.01–0.09 IU/ml), and full protective (> 0.1 IU/ml). The description of IgG titers in each group can also be seen based on the geometric mean titre (GMT).

**Table 1:** Characteristics of sample and antibody titer seroconversion (IgG) based on age, sex and residency

Variable	n	f (%)	Titer IgG			

			< 0.01 IU/ml (%)	0.01–0.09 IU/ml (%)	> 0.1 IU/ml (%)	GMT (IU/ml)
Age (YO)						
Young adults (16–20)	20	6.8	1(5.0)	4(20.0)	15(75.0)	0.19
Adults (21–60)	224	75.9	46(20.5)	76(33.9)	102(45.5)	0.06
Elderly (> 60)	51	17.3	10(19.6)	24(47.1)	17(33.3)	0.06

Gender						
Male	132	44.7	29(22.0)	49(37.1)	54(40.9)	0.05
Female	163	55.3	28(17.2)	55(33.7)	80(49.1)	0.07

Residency						
East Java	148	50.2	30(20.3)	54(36.5)	64(43.2)	0.06
Central Java	147	49.8	27(18.4)	50(34.0)	70(47.6)	0.06

Total	295	100	57 (19.3)	104 (35.3)	134 (45.4)	0.06

Table 1 shows there is a decreasing amount in the proportion of samples with full protective immune status (>1 UI/ml) occurred by age, although the difference in GMT values was only seen between the ages of 16–20 years compared to the other two age groups. Further, the difference in the proportion of full protective immune status between men and women and residency are unclear.

**Figure 2:**
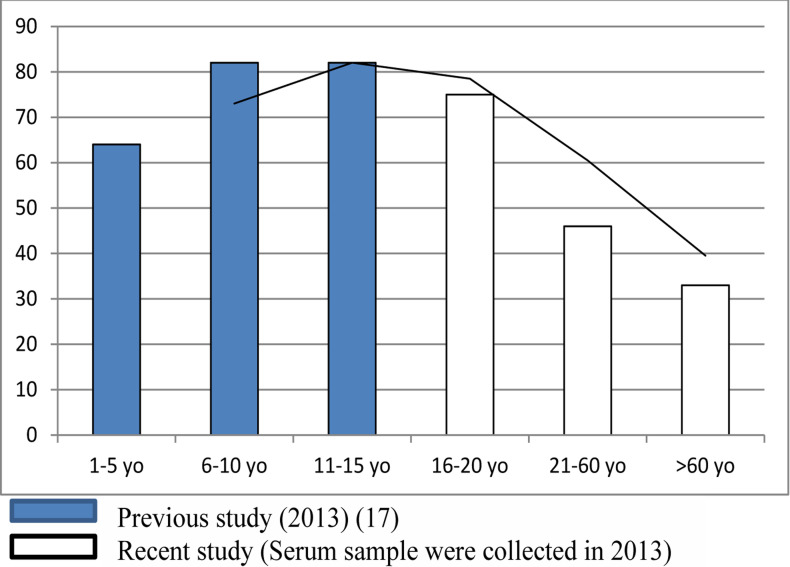
the rising and decreasing levels trends of immunity against diphtheria by age group of respondents. The data were merged with the results of previous studies in East Java in 2013 (17).

Changes in the proportion of samples with full protective immune status by age group from birth to elderly (> 60 years) are obviously noticed when the data are opposed by the results of previous studiy conducted in 2013 (Figure 2)^17^. The previous study was conducted only in 1 province (East Java) with a sample size which is almost the same and the same IgG examination method, neutralization.

Table 1 and Figure 2 shows that the proportion of samples with full protective immune status remained quite high (75%) in the age group of 16–20, but declined quite seriously (45.4%) in the age group of 21–60 and kept declining (33.3%) in the age group of elderly.

### Statistical analysis

Statistical analysis were carried out to confirm the significance of age, sex and residency on immune status against diphtheria (Table 2).

**Table 2:** The significance of age, sex and residency on immunity against diphtheria

Variable	>0.1 IU/ml (%)	OR	*p*-value
Age (years old)			
Young adults (16–20)	15 (11.2)		0.003
Adult (21–60)	102 (76.1)	0.6	
Elderly (> 60)	17 (12.7)	0.4	

Gender			
Male	54(40.3)	0.8	0.16
Female	80(59.7)		

Residency			
Central Java	70(52.2)	1.1	0.43
East Java	64(47.8)		

Table 2 shows that statistically only the factor of age impacted the immune status of diphtheria. Gender and residency have no impact to the immune status.

## Discussion

The samples came from 2 neighboring provinces, but the number of reported cases were extremely distant (Figure 1). Although, by percentage, the full protective immune status was higher in samples from Central Java, the value of GMT (Table 1) and statistical test results (Table 2) showed that the differences in percentage were not significant. Samples were not selected proportionally by district. It predicted influence the result which is different to the previous study, considering that prevalence of diphtheria in each district were different^17^,^19^. The data was not analyzed by immunization history and history of the exposure to the diphtheria cases because of data limitation. A history of vaccination will affect immune status. Likewise, a history of exposure to disease agents will also affect the maintenance of immunity in the natural state^17^–^19^. The interesting concern found here was when data of full protective immune status are categorized into two groups (not shown in the result), that were full protective (0.1–0.9 IU/ml) and long term protective (> 1 IU/ml). In East Java with more cases reported, the proportion of long term protective immune status (> 1 IU/ml) is higher than in Central Java (10.5%: 7.5%). In other hand, the proportion of full protective immune status (0.1–0.9 IU/ml) is higher in Central Java compared to East Java (39.9%: 33.3%). Two studies conducted by Hughes, et al. and Husada, et al in low cases district (Kediri) and high cases district (Bangkalan) also found out the same pattern^17^,^19^.

Table 1 also shows how the correlation between gender and immunity status. In percentage and GMT values (Table 2), it can be seen that both the proportion of full protective immune status and the higher mean titer IgG were found in women. Overall, the results of this study were similar to the previous studies in other countries, it showing us there was no difference between men and women for the immune status^20^. However, by percentage and GMT, these results were slightly different from the theory that seroprotective level of diphtheria tended to be higher in males than females^21^.

Out of the 3 factors analyzed, age factor was the only factor affecting the respondent's immune status (Tables 1 and 2). This was an evidence of the proportion of full protective and GMT immunity status. There was a decreasing in IgG titer as the age increasing. The most significant loss (full protective status and GMT) could be seen in the age group of 21–60 years old, compared to any age group (Figure 2). More detailed analysis with a range of 5 years in the age group of 21–60 years old (results not shown) revealed insignificant change of the immune proportion. It predicted relate to a continuous diphtheria vaccination program (booster) for every 10 years in adult, which has not been taken yet as a policy by Indonesian Government. Tomovici, et al showed that the peak of GMT IgG titers in diphtheria occurred about 1 month post-immunization and decreased annually and the lowest point caught after 10 years^18^. The result of this study is similar to the previous study. Zasada, et al. reported that antibody titer (IgG) against diphtheria in the Polish population also decreasing with age. However, a decrease in the proportion of immune status with IgG titer >0.1 IU/ml in Poland occurred in the age group over 40 years old. This is because Poland gave the last booster of diphtheria at the age of 19 years old^22^. Similar to the study of Zasada, et al., the decline in diphtheria immunity in European countries generally occurs at older ages^23^–^25^. As we know, most European countries apply the diphtheria vaccine booster policy for every 10 years^12^. In a developed country like Singapore, it was similar, the declining begun at older ages^26^. As previously stated, the mean of DPT3 vaccination coverage in Indonesia is 82%. The results showed that the proportion of individuals with full protective immunity can still be maintained (75%) up to the age group of 16–20 years old, but drastically decreased in the age group after. The coverage of 82% (79–84%) with a proportion of protective individuals 75% (74–78%) is a critical factor for obtaining a 75–80% diphtheria immunity threshold^27^. It shows the importance of running the continuous vaccination programs diphtheria in adults by Indonesian government.

The main limitation of the study is the unavailability of the vaccination history. This study also has limitations in number of samples and location. This is a preliminary study that requires further study with a large number of samples to represent Indonesia nationally.
